# Efficacy of quercetin derivatives in prevention of ulcerative colitis in rats

**DOI:** 10.2478/intox-2013-0002

**Published:** 2013-03

**Authors:** Ruzena Sotnikova, Viera Nosalova, Jana Navarova

**Affiliations:** Institute of Experimental Pharmacology and Toxicology, Slovak Academy of Sciences, Dubravska 9, 841 04 Bratislava, Slovak Republic

**Keywords:** quercetin derivatives, ulcerative colitis, intestinal damage

## Abstract

Reactive oxygen species has been implicated to contribute significantly to tissue injury associated with ulcerative colitis. Thus compounds with antioxidant properties could be potential therapeutic agents in this disease. Flavonoid compounds are known to possess antioxidative and antiinflammatory properties. Two derivatives of the flavonoid quercetin (Q), chloronaphthoquinone quercetin (CNC) and monochloropivaloyl quercetin (MCP), showed improved antioxidant properties and moreover, they efficiently inhibited aldose reductase activity *in vitro*. The aim of the work was to test the potential efficacy of quercetin and these synthetic derivatives in vivo in prevention of intestinal inflammation during ulcerative colitis in rats. Colitis was induced by intracolonic administration of acetic acid (4% solution). The control group received the same volume of saline. The vehicle dimethyl sulfoxide (DMSO) and the drugs Q, CNC or MCP were administered orally two hours and then one hour before the acetic acid or saline instillation. After 48 hours, the animals were sacrificed and the colon was weighed, measured and scored for visible damage. Acetic acid triggered an intense inflammatory response of the colon, characterised by haemorrhage, ulceration and bowel wall thickening. From the drugs tested, only CNC (2 × 50 mg/kg) effectively depressed inflammatory damage of the colon. The mechanism of this beneficial effect remains to be elucidated.

## Introduction

Ulcerative colitis is a chronically recurrent inflammatory bowel disease (IBD). Its aetiology however remains essentially unknown and the pharmacological treatment is still unsatisfactory, despite the great deal of attention it has received during the past years. It is now accepted that reactive oxygen species (ROS) are produced in excess by the inflamed mucosa and may play an important role in IBD etiopathogenesis (Loguercio *et al.*, 1996). Under physiological conditions, colonic mucosa contains relatively low tissue levels of endogenous antioxidants and oxidative stress may easily overwhelm the endogenous defence systems regulating ROS production. In cells from IBD patients, ROS are produced in abnormally high levels (Rezaie *et al.*, 2007), which leads to oxidative stress and thus to DNA damage due to an imbalance between innate and exogenous antioxidants and ROS (Hemnani, 1998). Thus compounds with antioxidant properties could be potential therapeutic agents in IBD.

The acetic-acid-induced ulcerative colitis in rats is one of the widely used animal models of inflammatory bowel disease (*e.g.* MacPherson & Pfeiffer, 1978; Noa *et al.*, 2000; Nosalova *et al.*, 2007). This experimental model resembles human ulcerative colitis in histology, eicosanoid production and response to sulphasalazine (Keshavarzian *et al.*, 1990). Inflamed colonic mucosa in acetic-acid-induced colitis is also known to produce excess ROS, which can be reduced by antioxidants (Keshavarzian *et al.*, 1992).

Flavonoids are increasingly viewed as beneficial dietary components, considering their well-established antioxidant and antiradical properties. In addition, flavonoid compounds exert many biological effects, including enzyme inhibition (lipoxygenase, cyclooxygenase, nitric oxide synthase), immune cell modulation, etc. (Middleton, 2000). Quercetin (3,3’,4’,5,6-pentahydroxyflavone) (Q) is normally present in plants as a glycoside as quercitrin or rutin (Sánchez de Medina *et al.*, 2002). Quercetin belongs to the most potent scavengers of ROS, including superoxide, peroxyl, alkoxyl and hydroxyl radicals, and reactive nitrogen species like NO•. and ONOO– (Butkovic *et al.*, 2004; Amic *et al.*, 2007; Boots *et al.*, 2008). Its antioxidative properties were confirmed also in humans. Quercetin and other flavonoids dramatically reduced oxidative stress *in vitro* in lymphocytes from IBD patients and healthy individuals (Najafzadeh *et al.,* 2009). Moreover, quercetin and its derivatives were found to be inhibitors of the *Helicobacter pylori*-stimulated respiratory burst of neutrophils (Bonacorsi *et al.,* 2012) and noncompetitive urease inhibitors (Xiao *et al.,* 2012), indicating that they may be potentially useful in the therapy of other gastro-intestinal diseases, *e.g.* *Helicobacter pylori*-induced gastric ulcer.

A series of synthetic derivatives of quercetin was prepared to improve antioxidant and other beneficial properties of the flavonoid. Since aldose reductase is involved in the pathogenetic way of inflammation, its blockade should ameliorate tissue injury induced by inflammatory processes (Chang *et al.*, 2012). Quercetin derivatives were tested also for their inhibitory effect on aldose reductase. From the new derivatives of quercetin, chloronaphthoquinone quercetin and monochloropivaloyl quercetin showed improved antioxidant properties and moreover, they efficiently inhibited aldose reductase activity (Veverka *et al.*, 2013). In this work, a model of acetic-acid-induced ulcerative colitis in rats was used for testing the potential efficacy of these synthetic derivatives also under *in vivo* conditions in prevention of intestinal inflammation.

## Methods

Male Wistar rats (270–300 g) from the Breeding Facility of the Institute of Experimental Pharmacology and Toxicology Dobrá Voda (Slovak Republic) were used. Animal experiments were conducted under the guidelines of the Ethics Committee of the Institute of Experimental Pharmacology and Toxicology, Slovak Academy of Sciences and were approved by the State Veterinary and Food Administration of the Slovak Republic. The rats were maintained under a 12 h light/dark cycle with free access to water.

### Induction of colitis and assessment of damage

The 24-h-fasted rats were anaesthetised with thiopental (65 mg/kg i.p.). A rubber cannula (8 cm long) was inserted into the colon through the anus. Acetic acid (4% solution in water, 1.5 ml) was instilled and the cannula was left in the colon for 30 s and then the fluid was withdrawn. The control group (CONTROL) received saline. Two hours and then one hour before the acetic acid or saline instillation, the rats were administered orally saline (COLITIS group), the vehicle pure dimethyl sulfoxide (DMSO group), quercetin (group Q: 2 × 50 mg/kg), chloronaphthoquinone quercetin (group CNC 50: 2 × 25 mg/kg; group CNC 75: 2 × 37.5 mg/kg; group CNC 100: 2 × 50 mg/kg) or monochloropivaloyl quercetin (group MCP 100: 2 × 50 mg/kg). After 48 hours, the animals were sacrificed by exsanguination in thiopental (65 mg/kg i.p.) anaesthesia. The colonic segments, rinsed with cold saline, were placed on an ice-cold plate, cleaned of fat and mesentery, and blotted on filter paper. The colon was cut longitudinally and scored for visible damage according to Nosalova *et al.* (2007) by an observer unaware of the treatment. The following criteria were used for scoring colonic damage: 0 - normal appearance; 1 - hyperaemia; 2 - haemorrhage; 3 - one ulcer; 4 - two or more sites of ulceration; 5 - ulceration exceeding >1 cm along the length of the colon; 6 and further - damage exceeding >2 cm along the colon length, and with each additional centimetre of involvement the score was increased by one point. The colons were weighed and measured and the colonic wet weight/longitude was calculated.

The drugs were dissolved in concentrated DMSO. The volume of the drugs administered did not exceed 1 ml.

Dimethyl sulfoxide (DMSO) was purchased from AppliChem GmbH (Darmstadt Germany), quercetin from Sigma-Aldrich Co. (St. Louis, MO, USA) and quercetin derivatives chloronaphthoquinone quercetin and monochloropivaloyl quercetin were synthesised by Eurofins Bel/Novamann.

### Statistical analysis

All results are expressed as mean ± S.E.M. Differences among groups were tested for statistical significance using ANOVA with the posteriori Bonferoni test, nonparametric data were analysed by the Mann-Whitney U-test. Statistical significance was set at *p<*0.05.

## Results

**Figure 1:**
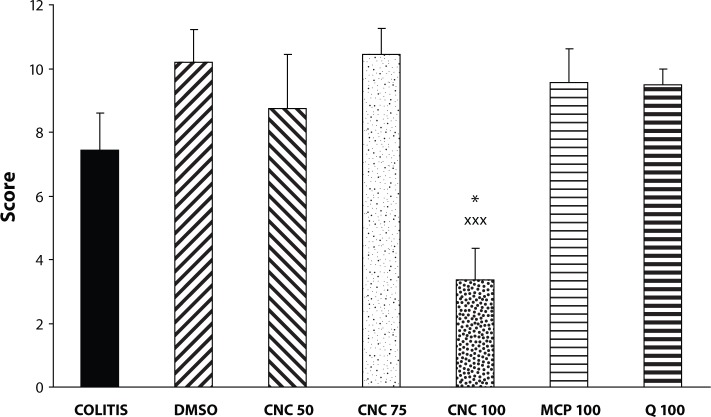
Effect of DMSO, quercetin (Q), chloronaphtoquinone quercetin (CNC 50: 2 × 25 mg/kg, CNC 75: 2 × 32.5 mg/kg, CNC 100: 2 × 50 mg/kg) and monochlorpivaloyl quercetin (MCP 100: 2 × 50mg/kg) p.o. on the damage score in acute experimental colitis induced in rats by 4% acetic acid. COLITIS – non-treated colitis. Results are mean ± S.E.M. of 8–12 experiments. Statistical analysis by Mann–Whitney U-test. *p<0.05 versus COLITIS, ^xxx^ *p<*0.001 versus DMSO.

**Figure 2:**
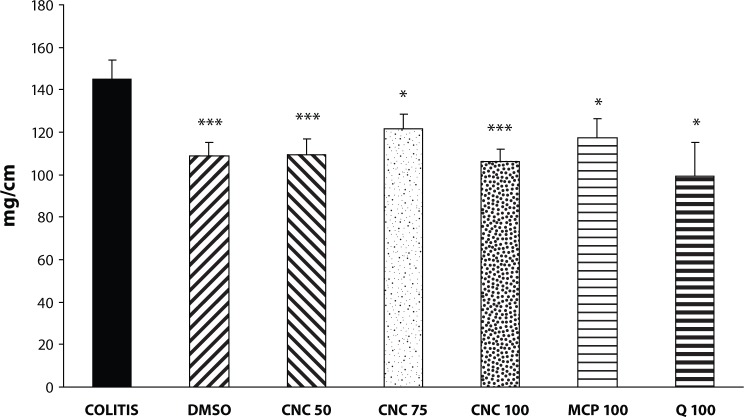
Effect of DMSO, quercetin (Q), chloronaphtoquinone quercetin (CNC 50: 2 × 25 mg/kg, CNC 75 – 2 × 32.5 mg/kg, CNC 100: 2 × 50 mg/kg) and monochlorpivanoyl quercetin (MCP 100: 2 × 50mg/kg) p.o. on the colonic wet weight/longitude ratio in acute experimental colitis induced in rats by 4% acetic acid. COLITIS – non-treated colitis. Results are mean ± S.E.M. of 8–12 experiments. Statistical analysis by ANOVA with Bonferoni posttest. *p<0.05 versus COLITIS, **p<0.001 versus COLITIS.

As expected, colonic instillation of acetic acid triggered an intense inflammatory response of the large bowel, characterised by extensive haemorrhage, occasional ulceration and bowel wall thickening. The damage score was 7.43±1.18. Administration of DMSO did not significantly influence acetic-acid-induced colonic injury (score 9.45±1.03). Equally, quercetin (2 × 50mg/kg) or MCP (2 × 50 mg/kg) treatment had no effect on macroscopic injury (score 9.50±0.50 and 9.57±1.04, respectively). From the three doses of CNC, only CNC 100 (the dose of 2 × 50 mg/kg) was found to be satisfactory in the protection of the rat colon against acetic-acid-induced injury – the damage score was 3.35±1.01 (Figure 1). The CNC 100 dose caused also reduction of the length of colonic injury from 70.77±7.91% to 44.19±5.60% (*p<*0.025). The colonic wet weight/length ratio increased in the COLITIS group from 76.13±4.37 to 145.01±8.87 mg/cm. The administration of DMSO as well as of all other treatments decreased this ratio, which however remained still significantly higher than that of the CONTROL group (Figure 2).

## Discussion

Our experiments showed that the chloronaphthoquinone derivative of quercetin in the dose of 2 × 50 mg/kg p.o. alleviated the manifestation of colonic inflammatory injury induced by acetic acid – it depressed the score as well as the length of the damaged colon. Quercetin in the same dose on the contrary, had no protective effect in our experimental model. It was reported that in *in vitro* conditions quercetin inhibited the production of inflammation-producing enzymes cyclooxygenase and lipoxygenase (Kim *et al.*, 1998; Lee *et al.*, 2010). It was found to inhibit TNF-α (Chuang *et al.*, 2010), nitric oxide production, and nitric oxide synthase expression (Ortega *et al.*, 2010). Some *in vivo* animal experiments supported also an antiinflammatory effect of quercetin (Morikawa *et al.,* 2003). Alarcón de la Lastra *et al.* (1994) found that oral pre-treatment with a high dose of quercetin (200 mg/kg) protected the rat gastric mucosa against ethanol-induced necrosis. Different results were found in studies in which the quercetin glycoside – quercitrin was tested for acute and chronic antiinflammatory activity in experimental models of colitis. Differences were found in the effective dose of the flavonoid tested. For instance, Sánches de Medina *et al.* (1996) reported that in the acute model of trinitrobenzenesulfonic-acid-induced rat colitis, quercitrin in the doses 1 or 5 mg/kg p.o. ameliorated colonic damage, yet it failed to attenuate the severity of the lesions. An increase or reduction of the dose of the flavonoid resulted in a marked loss of the effect. Also in the model of chronic ulcerative colitis induced by dextransodiumsulphate in rats (Camuesco *et al.*, 2004), the doses of quercitrin 1 or 5 mg/kg p.o. significantly attenuated the response compared to the non-treated control group. We suppose that disparities in effective doses of quercetin and quercitrin may be based on their different absorption profile. The assumption that the controlled release of quercetin would improve its therapeutic effect was confirmed in the model of acetic-acid-induced colitis by using quercetin-loaded microcapsules (Guazelli *et al.,* 2013).

One of the manifestations of inflammatory damage of the colon in ulcerative colitis in rats is bowel wall thickening. The relative wet weight of the inflamed colon tissue is considered a reliable and sensitive indicator of the severity and extent of the inflammatory response (Rachmilewitz *et al.,* 1989). In our experiments, acetic-acid instillation induced an increase in the relative weight of the colon. Administration of the vehicle DMSO reduced this parameter significantly, which was however not further influenced by the other drugs tested. Thus, it is clear that quercetin and its derivatives in the doses studied are either ineffective or less effective than DMSO in prevention of colon wall thickening during inflammation. DMSO was reported to have many different beneficial effects (for review see Santos *et al.*, 2003; Jacob and de la Torre, 2009). Some of them, *e.g.* hydrogen radical scavenging properties and reduction of platelet adhesion by collagen, may presumably be involved in the inhibition of bowel wall thickening in colitis.

In conclusion, in the model of acetic-acid-induced colitis in rats, chloronaphthoquinone derivative of quercetin in the dose of 2 × 50 mg/kg p.o. effectively depressed inflammatory damage of the colon. The question whether its antioxidant activity, inhibition of aldose reductase or other properties are responsible for this beneficial effect remains still open.
